# Endoscopic Ultrasound-Guided Radiofrequency Ablation for an Adrenocorticotropic Hormone-Secreting Pancreatic Neuroendocrine Tumor on Antiplatelet Therapy

**DOI:** 10.14309/crj.0000000000002374

**Published:** 2026-10-28

**Authors:** Olivia Langton, Sai Sudha Valisekka, Rahul Karna, Guru Trikudanathan

**Affiliations:** 1 Department of Internal Medicine, University of Minnesota, Minneapolis, MN; 2 Division of Gastroenterology, Hepatology and Nutrition, Department of Medicine, University of Minnesota, Minneapolis, MN

**Keywords:** radiofrequency ablation, endoscopic ultrasound, pancreatic neuroendocrine tumor

## Abstract

Endoscopic ultrasound (EUS)-guided radiofrequency ablation (RFA) has been used for thermal destruction of precancerous insulinomas and nonfunctioning pancreatic neuroendocrine tumors (pNETs) by coagulative necrosis. We report a case of a critically ill patient with an adrenocorticotropic hormone (ACTH)-secreting pNET treated with EUS-guided RFA (EUS-RFA) while on a P2Y12 antiplatelet after temporary stabilization on a titratable continuous etomidate infusion. This case demonstrates the novelty of EUS-RFA as a procedural treatment option for functional pNETs in critically ill, nonsurgical candidates on antiplatelet therapy.

## INTRODUCTION

Radiofrequency ablation (RFA) is a technique used to thermally destroy targeted tissue by releasing high frequency energy, often performed via endoscopic ultrasound (EUS) to treat pancreatic lesions.^1^ The technique is considered less invasive than surgical resection, and hence, more feasible in high-risk patients.^2^ Pancreatic neuroendocrine tumors (pNETs) are a heterogeneous group of neoplasms that can be functional (hormone-secreting) or nonfunctional, with the majority (∼70%) of pNETs being nonfunctional.^1,3^ Of the functional subset, the most common variants are insulinomas and gastrinomas.^3^ Adrenocorticotropic hormone (ACTH)-producing pNETs are rare but are associated with a downstream increase in cortisol production.^4^ To our knowledge, this is the first documented case of endoscopic ultrasound-guided radiofrequency ablation (EUS-RFA) for a functional ACTH-secreting pNET in a critically ill, intubated patient performed on antiplatelet therapy.

## CASE REPORT

A 63-year-old woman presented to her primary care provider with worsening hyperglycemia, bloating, and resistant hypertension. Laboratory workup ultimately confirmed ACTH-dependent Cushing syndrome. Computed tomography abdomen demonstrated a 3.5 × 2.4 cm solid lesion distinct from the pancreatic head without ductal dilatation or associated inflammatory changes (Figure 1). EUS-guided biopsy confirmed a well-differentiated pNET that stained positive for ACTH. Magnetic resonance imaging of the pituitary gland ruled out a pituitary mass but revealed acute and subacute infarcts. Her hospital course was complicated by extensive pulmonary emboli and Rhodococcus cavitary pneumonia. She required thrombectomy, carotid stenting, and treatment with ticagrelor and heparin. She was started on oral metyrapone and ketoconazole to inhibit steroidogenesis and block excess cortisol production and was admitted to the intensive care unit for intubation. The pharmacokinetics of metyrapone prevented crushing for administration via nasogastric tube, so a titratable continuous intravenous (IV) etomidate infusion was started to maintain cortisol levels ∼20 mcg/dL. Within 72 hours, her cortisol levels decreased from 81.1 mcg/dL to 17.1 mcg/dL. However, ketoconazole was paused due to worsening transaminases (alkaline phosphatase 211 U/L, aspartate aminotransferase 169 U/L, and alanine aminotransferase 108 U/L).

**Figure 1.**
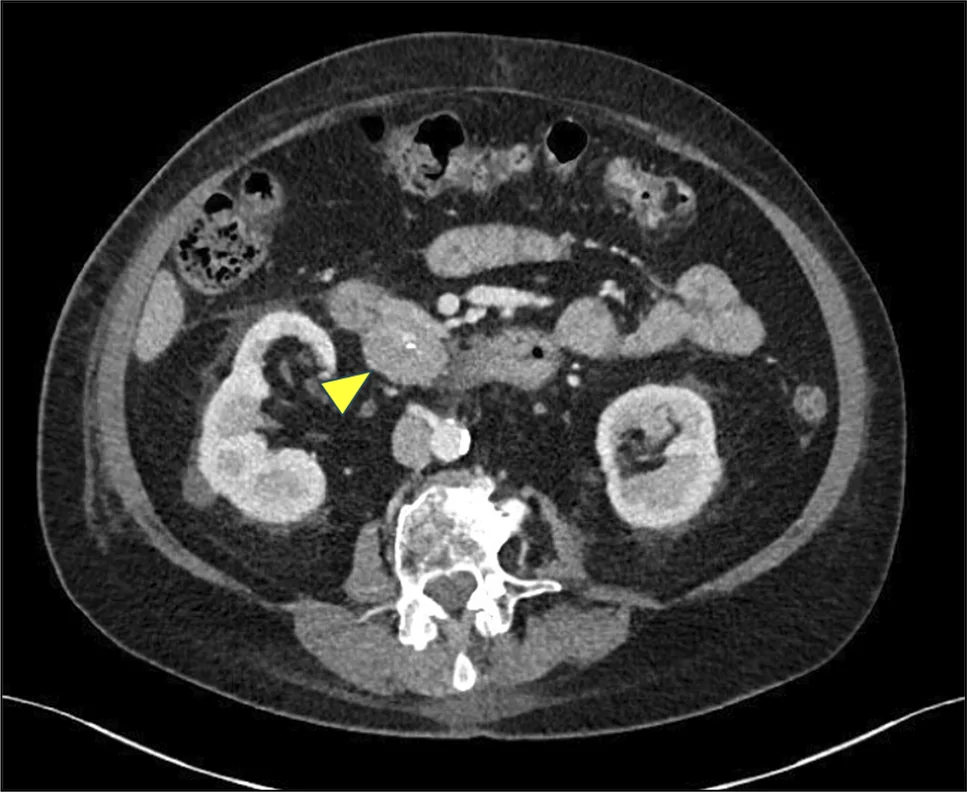
Computed tomography visualization of the 3.5 × 2.4 cm solid lesion extending from the pancreatic head without pancreatic ductal dilatation or associated inflammatory changes.

Subsequently, the patient was deemed a high-risk candidate for surgical resection of the pNET given her comorbidities. Transarterial embolization was considered to functionally disrupt the blood supply, but the tumor was poorly located for percutaneous access and there was a very high chance of precipitating severe pancreatitis. EUS-guided ethanol ablation (EUS-EA) was considered, but there was a high risk of precipitating necrosis in the setting of antiplatelet therapy. After a multidisciplinary meeting, EUS-RFA was pursued, while the patient was continued on ticagrelor with a temporary pause of the heparin drip.

EUS visualization of the tumor demonstrated a 37 × 33 mm pancreatic lesion on the inferior head of the pancreas (Figure 2). Borders were well-defined but irregular with a 6 mm shadowing calcification and multiple large caliber feeding vessels. A 19-gauge EUS-RFA probe (Starmed; Taewong, South Korea; 19-10 E; tip length 5 mm) was advanced into the lesion (Figure 2). Ablation energy was emitted at 30 W for 24 cycles, with each cycle performed until visualization of hyperechoic bubbles was achieved (Figure 2). Timing ranged from 8-19 seconds per cycle. After the procedure, an estimated 70%–80% of the tumor was successfully ablated (Figure 2). Given the significant risk of bleeding on ticagrelor, the remaining portion adjacent to the vasculature was avoided and no bleeding was observed. Within 12 hours post-RFA, etomidate was discontinued, and low-dose ketoconazole was restarted with serum cortisol maintained at ∼30 mcg/dL.

**Figure 2.**
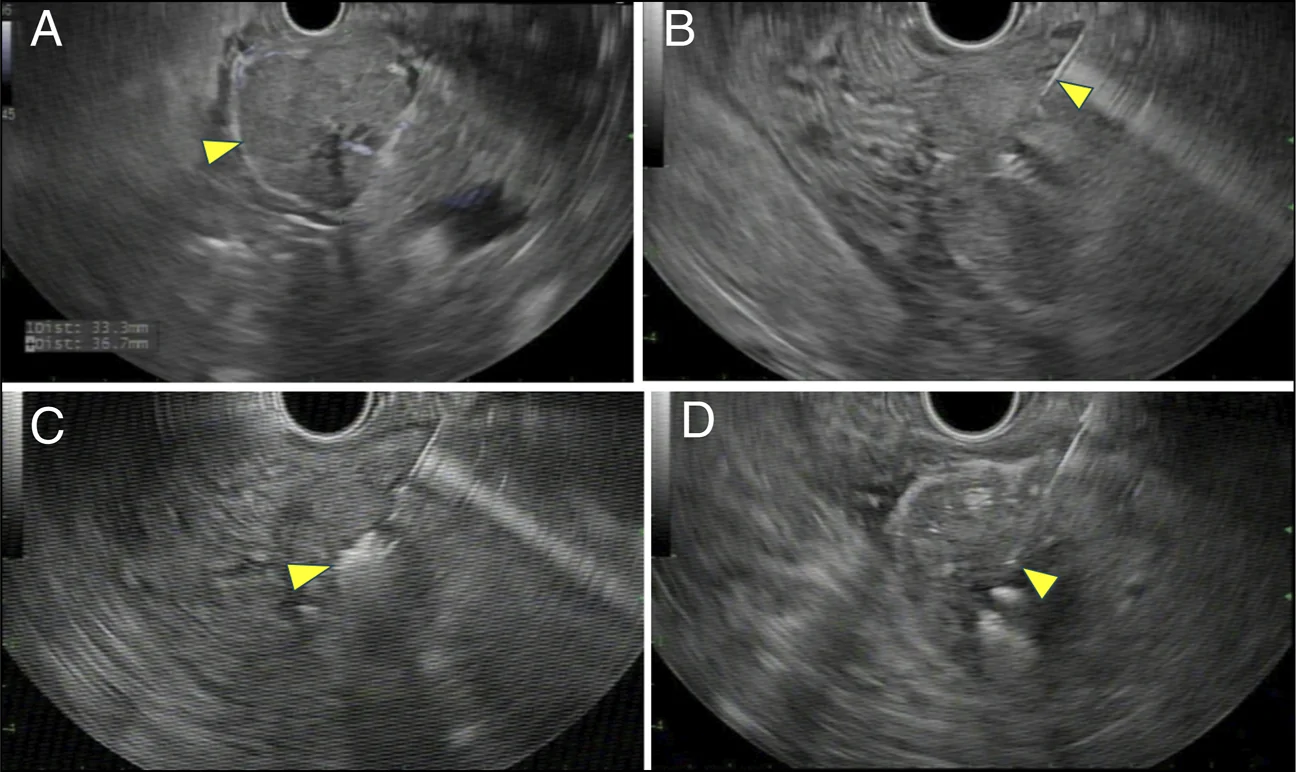
A, Endoscopic ultrasound visualization of the pancreatic neuroendocrine tumor before radiofrequency ablation (RFA). B, RFA probe located within the pancreatic neuroendocrine tumor. C, Visualization of the hyperechoic bubbling, the end point of each ablation cycle. D, RFA probe located within the pancreatic neuroendocrine tumor after 24 cycles.

Around 24 hours after RFA, the patient was extubated. Hemoglobin remained stable at 8.1 g/dL, and no melena or hematochezia was observed. Additional RFA sessions were initially planned to ensure adequate disease control, but her mental status failed to recover, and her family made the decision to transition to comfort care. The patient passed away from the sequelae of her cerebral infarcts 3 days later.

## DISCUSSION

ACTH-producing pNET are rare functional pNETs that induce hypercortisolism leading to downstream hormonal effects and endogenous Cushing syndrome.^4^ Management of functional pNETs is centered around cessation of symptoms from excessive hormone production,^1^ in this case, ectopic ACTH-induced production of cortisol. Medical management is often necessary as the first step to bridge patients to definitive intervention.^5^ Our case demonstrates that patients who are high risk for surgical resection may undergo EUS-RFA for management.

Medications such as metyrapone, osilodrostat, and ketoconazole can be used to inhibit steroidogenesis, including blocking 11β-hydroxylase and 17α-hydroxylase, to disrupt cortisol production.^6^ Etomidate has also been used as an effective therapeutic modality when other medications are contraindicated or unavailable, more often in critically ill patients, as it is the single available intravenous option to disrupt the cortisol production pathway.^5,7^ Previously, Krzystek et al^8^ reported a patient with an ACTH-secreting pNET who was started on a continuous low-dose etomidate infusion to rapidly lower cortisol levels, then initiated on osilodrostat for suppressive therapy. The patient was weaned off etomidate and maintained on osilodrostat for 19 months prior to intervention with EUS-EA. While oral medications can be effective treatments for hypercortisolism, they could not be used in our patient due to her critically ill state. The patient's tenuous clinical status led to the necessary use of etomidate to bridge the patient to EUS-RFA with the goal of extubation and stabilization for future pancreaticoduodenectomy.^9^

To definitively eliminate the excess hormone, the neuroendocrine cells responsible for the hyperproduction must be destroyed or removed.^2^ The most common nonpharmacological modalities of treatment include surgical resection, EUS-RFA, and EUS-EA.^1,10–12^ Surgery is often the treatment of choice as recommended by the National Comprehensive Cancer Network (NCCN) Neuroendocrine and Adrenal Tumors Guidelines when a pNET is localized, without distant metastases, and without local invasion.^9^ In this case, after a multidisciplinary meeting, given the lack of surgical options or radiology guided ablative techniques, EUS-RFA was chosen as the most feasible, least invasive procedural modality with the goal of ablating enough tissue to discontinue the etomidate and stabilize the patient for eventual pancreaticoduodenectomy.

EUS-RFA is a feasible modality for ablation of pNETs, with low recurrence and adverse event rates.^2^ Prior studies have demonstrated EUS-RFA as an effective modality for management of functional pNETs that involve insulinomas.^3,13^ Previously, a retrospective analysis of 7 patients with insulinoma undergoing EUS-RFA demonstrated complete normalization of glucose within 24 hours of EUS-RFA in all 7 patients with no clinically significant effects at follow-up 2-21 months later.^14^ There are limited data on EUS-RFA for ACTH-secreting pNETs. Sideri Gugger et al^15^ reported on the successful ablation of a small, 9-mm ACTH-secreting pNET of the pancreatic tail in a noncritical but nonsurgical candidate. Given the size of the pNET, the procedure required only 1 session of 3 RFA treatments (emitted at 10 W) with a 19-gauge probe. In contrast, our patient had a tumor ∼4 times larger (37 × 33 mm), requiring 24 cycles of ablation (emitted at 30 W) with plans for additional sessions. Our case was then further complicated by the patient's antiplatelet therapy which could not be held prior to the procedure. To the best of our knowledge, this is the first documented case of EUS-RFA for an ACTH-secreting pNET in a critically ill, intubated patient performed on antiplatelet therapy.

This case highlights the novelty of EUS-RFA as an intervention for functional pNETs and further highlights the rarity of ACTH-secreting pNETs. It also demonstrates the expanding use of this technique beyond stable patients and demonstrates that EUS-RFA is feasible in critically ill patients on continued antiplatelet agents when these medications cannot be safely discontinued. However, as a single case, these findings are limited by generalizability and conclusions regarding efficacy and safety cannot be drawn. Furthermore, the procedure was only able to successfully ablate 70%–80% of the tumor, potentially confounding the outcomes, which were further complicated by the resumption of ketoconazole 3 days after EUS-RFA. Although promising, without pathological confirmation of ablated tissue and long term follow-up data, the outcome of the EUS-RFA cannot be attributed to the procedure alone, and further research is needed.

## DISCLOSURES

Author contributions: O. Langton: first author, drafted and edited manuscript. S.S. Valisekka: guided discussion structure and edited manuscript. R. Karna: edited and guided manuscript. G. Trikudanathan: conceptualization, reviewed draft, supervised, and guided manuscript. G. Trikudanathan is the article guarantor.

Financial disclosure: G. Trikudanathan is a Consultant for Boston Scientific, Research funds from AbbVie Inc.

Previous presentation: This case was presented at Endocrine Society (ENDO) 2026, June 13-16, 2026; Chicago, IL.

Informed consent was obtained from the patient's family for this case report.

## ABBREVIATIONS:

ACTH: adrenocorticotropic hormone
ENDO: Endocrine Society
EUS: endoscopic ultrasound
EUS-EA: endoscopic ultrasound-guided ethanol ablation
EUS-RFA: endoscopic ultrasound-guided radiofrequency ablation
NCCN: National Comprehensive Cancer Network
pNETs: pancreatic neuroendocrine tumors
RFA: radiofrequency ablation
